# Cooling wound dressings: Prospects for clinical practice

**DOI:** 10.1002/ctm2.70064

**Published:** 2024-10-18

**Authors:** Peng Chen, Pingping Zhang, Jiangang Sun, Yangzhe Hou, Xianhu Liu

**Affiliations:** ^1^ Department of Gastrointestinal Surgery The First Affiliated Hospital of Zhengzhou University Zhengzhou China; ^2^ Department of Infectious Diseases Children's Hospital Affiliated to Shandong University Jinan China; ^3^ National Engineering Research Center for Advanced Polymer Processing Technology Zhengzhou University Zhengzhou China; ^4^ UniSA STEM and Future Industries Institute University of South Australia Adelaide South Australia Australia

## 1 INTRODUCTION

The skin, as the largest organ of the human body, is often compromised by trauma, severe burns, ulcers and various other injuries.^1^, ^2^ Such injuries not only precipitate pain and scarring but also significantly increase the risk of microbial infection. Consequently, there is an urgent imperative to develop effective treatment strategies to address these concerns. Wound dressings serve as crucial therapeutic tools that safeguard wounds, decrease the likelihood of contamination and infection, and promote tissue repair by absorbing exudates and maintaining a local moist environment. In recent years, a variety of functional polymer‐based medical dressings have been developed – including polymer films.^3^, ^4^ hydrogels.^5^, ^6^ sponges and foams.^7^, ^8^ demonstrating excellent biocompatibility and significant potential for application in skin tissue engineering. These biocompatible dressings are characterised by antibacterial, antioxidant and controlled drug release properties. Additionally, they can function as flexible electrodes for electrotherapy and facilitate real‐time monitoring of wound status, thereby enhancing their value in clinical applications.

Nevertheless, in the development of multifunctional wound dressings, it is imperative to emphasise their fundamental roles in wound protection and environmental regulation, particularly the regulation of wound temperature, which is of paramount importance.^9^, ^10^, ^11^ Wounds are often accompanied by inflammation and increased localised temperature, while patients may also experience occasional exposure to direct sunlight and heat. Excessive heat can worsen tissue damage, raise the risk of infection, and slow healing. It may also promote fibroblast tissue overgrowth, increasing the likelihood of scarring. While conventional wound dressings are effective in protecting wounds and promoting healing, they still exhibit notable limitations in temperature regulation.^12^, ^13^, ^14^

Passive radiative cooling (PRC) works by reflecting sunlight at wavelengths of 0.3 to 2.5 µm and utilising the atmospheric long‐wave infrared transmission window (8 to 13 µm) to dissipate excess heat into outer space.^15^, ^16^, ^17^ This process occurs without the need for mechanical equipment or energy consumption. Cooling materials with PRC properties can be engineered in various forms and structures to meet diverse application needs. Recent cooling research has also increasingly focused on biodegradable materials with improved biocompatibility.^18^, ^19^ making the integration of PRC properties into medical wound dressings a highly promising development. This noninvasive approach optimises clinical treatment by utilising physical cooling mechanisms. It also serves as a carrier for slow‐release medications, providing both bacteriostatic effects and highly effective wound healing promotion. Consequently, cooling wound dressings are anticipated to be an optimal choice for postsurgical care, chronic wound treatment, and specialised wound management, including burn care.

## 2 FUNCTIONAL POLYMER‐BASED MEDICAL WOUND DRESSING

**FIGURE 1:**
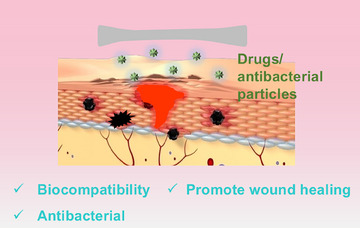
Common antimicrobial wound dressings.

Advancements in science and technology have led to a growing demand for functionality in wound dressings, with antibacterial properties being especially critical. Although materials such as chitosan (CS) exhibit some broad‐spectrum antibacterial effects, their efficacy is limited and can be enhanced through the incorporation of nanomaterials or drugs to bolster their antibacterial properties (Figure 1).^3^, ^5^ It is noteworthy that Yuan's research team has obtained a series of significant results regarding the efficient and broad‐spectrum antibacterial effects of metal nanoclusters. For instance, they synthesised light‐driven antimicrobial agents by combining photoluminescent gold nanoclusters (AuNCs) with titanium dioxide (TiO₂). The Au NCs function as photosensitisers, effectively promoting the segregation of photogenerated carriers, which enhances the production of reactive oxygen species and efficiently eliminates bacteria.^20^ Furthermore, the team encapsulated ultrasmall silver nanoclusters onto the bacterial medium using light irradiation, thereby further enhancing antibacterial activity.^21^

Ideal wound dressings should provide not only antibacterial property and healing of host tissue but also external protection. As mentioned earlier, to enhance their clinical efficacy, nanomaterials often need to be incorporated into an appropriate matrix. Yuan's research team also developed an injectable wound dressing composed of Ag_29_ nanoclusters and mangiferin molecules integrated with CS hydrogel. This dressing exhibited excellent biocompatibility and broad‐spectrum antimicrobial properties, effectively promoting cell proliferation and demonstrating significant potential for preventing infection and accelerating wound healing.^22^

Moreover, the conductivity of wound dressings is emerging as a prominent research topic. Conductive polymer dressings, developed through advanced processing design, exhibit significant application potential due to their electrotherapeutic efficacy and capability for real‐time monitoring of wound status. Long et al.^23^ developed a highly efficient electric bandage that employs a nanogenerator to convert mechanical displacements from skin movement into electrical energy, thereby generating an alternating electric field. This electric field has been shown to effectively promote the migration, proliferation, and differentiation of fibroblasts, thereby accelerating the healing process of skin wounds. Bhang et al.^24^ engineered a piezoelectric skin patch comprising unidirectionally aligned zinc oxide (ZnO) nanorods, which generate piezoelectric potentials in response to mechanical deformations induced by animal movement. This phenomenon creates an electric field at the wound site, thereby enhancing cellular metabolism, migration, and protein synthesis, ultimately facilitating the acceleration of wound healing.

The aforementioned studies illustrate the development of functional innovations in medical wound dressings; however, for clinical practical applications, these dressings must also possess good breathability and cell adhesion properties. Therefore, aerogels, porous films and fibrous membranes exhibit promising applications in wound dressings and skin scaffolds. For instance, Karahaliloglu et al.^25^ successfully utilised CS/silk protein scaffolds combined with lauric acid and ZnO nanoparticles in wound repair dressings, demonstrating significant antimicrobial effects against *Escherichia coli* and *Staphylococcus aureus*. Lanno et al.^26^ fabricated porous Polycaprolactone microfibre scaffolds using electrostatic spinning in a high‐humidity environment, demonstrating that these scaffolds exhibited superior performance in promoting cell attachment and growth compared to nonporous fibres. These studies further indicate that porous structures play a crucial role in promoting wound healing, highlighting their significant potential in clinical diagnosis and treatment.

## 3 TRANSITIONING FROM BIO‐BASED PRC MATERIALS TO COOLING WOUND DRESSINGS IN CLINICAL PRACTICE

As shown in Table 1, recent years have seen increased attention on research involving bio‐based PRC materials. For example, Xu et al.^27^ developed composite hydrogels with sustainable passive cooling by incorporating zirconium dioxide (ZrO_2_) particles, lithium bromide (LiBr) as a hygroscopic agent, and polyacrylamide (PAAm). These hydrogels achieved average temperature reductions ranging from 3.2°C to 5.0°C under intense sunlight on the first day, depending on the LiBr loading ratios. By compounding TiO₂ with polylactic acid (PLA), Zeng et al.^28^ developed a cooling material that could reduce temperature by approximately 4.8°C compared to commercial cotton fabrics when applied to the human body. Deng et al.^29^ on the other hand, designed a hybridised aerogel cooler composed of CS, aluminium chloride (AlCl_3_), a polyvinyl alcohol (PVA) network, and silica sol, achieving a temperature reduction of over 9°C compared to ambient conditions under direct sunlight. Our research team is also focused on the design of porous PLA materials, which exhibit excellent biocompatibility and radiative cooling properties. For instance, we have developed porous PLA membranes that combine flexibility with high porosity (90.1%),^30^ along with PLA aerogels demonstrating significant cooling effects (3.5°C reduction under daytime conditions).^18^

**TABLE 1:** Biocompatible and biodegradable PRC materials.

Cooling materials	Daytime cooling	Night‐time cooling	Comparison
DNA and gelatin layered aerogel^31^	16.0°C	1.6°C	Ambient temp.
PLA aerogel with hierarchical structure^18^	3.5°C	5.8°C	Ambient temp.
PLA‐TiO_2_ composite fabrics^28^	4.8°C	–	Commercial cotton fabrics
PAAm/ ZrO_2_/LiBr composite hydrogel^27^	3.2°C–5.0°C	–	Ambient temp.
CS/SiO_2_ Composite Fabrics^32^	11.2°C	5.4°C	Commercial cotton fabrics
Heterogeneous aerogel composed of CS/AlCl_3_/PVA networks and silica sol^29^	9.0°C	–	Ambient temp.
Cellulose nanofibre and SiO_2_ composite paper^33^	3.0°C	–	Ambient temp.
Porous cellulose acetate membrane^34^	4.6°C	2.9°C	Ambient temp.
Natural silk fibre electrospun film^35^	7.5°C	–	Nonwoven raw silk fabric
Biomass‐derived silk fibroin/PLA fibre membrane^36^	6.0°C	–	Ambient temp.

Recently, *Science* reported on a layered photoluminescent aerogel composed of DNA and gelatin.^31^ This aerogel is not only repairable and biodegradable but also scalable through water welding. Under high‐intensity solar irradiation, it can reduce the ambient temperature by 16.0°C. Based on this innovation, our team prospects the significant potential of the next generation of green biodegradable radiative cooling materials for environmental protection and energy‐saving applications.^37^ Currently, related research concentrates on developing biodegradable building materials and wearable fabrics that mitigate the environmental impact of global warming and petroleum‐based polymers through green, energy‐free cooling. In contrast, the potential of bio‐based PRC materials for clinical applications, particularly in cooling wound dressings, remains largely underexplored.

Intriguingly, Zhu and colleagues recently reported in *Nature Chemical Engineering* the development of a polyamide 6/silk fibroin bilayer dressing designed for sunlight‐exposed wound healing.^38^ This innovative dressing exhibits high mid‐infrared emissivity (0.94) and sunlight reflectivity (0.96), enabling it to maintain a temperature approximately 7°C lower than the ambient temperature in direct sunlight. Additionally, its optimised layered nanofibre structure ensures excellent moisture permeability while providing effective protection against external bacteria. Building on this work, Tao's research team has further highlight that this first application of PRC cooling technology in wound dressings marks a significant milestone in the field.^39^ However, this field is still in its infancy, and further research is urgently needed to address these gaps.

**FIGURE 2:**
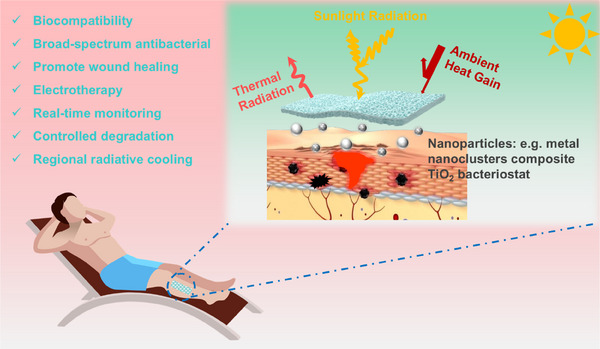
New generation multifunctional integrated dressings.

Undoubtedly, broad‐spectrum antimicrobial agents based on metal nanoclusters complexed with TiO₂.^20^ along with conductive dressings incorporating nanofillers (e.g., Ag and ZnO nanoparticles).^4^, ^24^ have demonstrated significant potential for clinical wound dressing applications. On the other hand, the high solar reflectivity and infrared emissivity needed for radiative cooling can be enhanced through the incorporation of nanomaterials and by designing with multistage pore structures. Thus, these functional properties can be optimally integrated through polymer processing to develop high‐performance cooling wound dressings. This ensures that the patient's wound area stays cool for extended periods, even in hot outdoor environments. Additionally, some biocompatible materials, like PLA, can have their degradation rate controlled by adjusting their crystallisation behaviour.^40^ This property can be utilised to create wound dressings that do not require removal or recycling. By combining superior antimicrobial activity, electrical conductivity, temperature‐reducing capability, and other special features, these dressings will offer more effective strategies for clinical wound management (Figure 2).

## 4 CONCLUSIONS AND PROSPECTS

With advancements in medical technology, wound dressings are drawing increasing attention from researchers and clinicians as an emerging therapeutic tool. Current studies suggest that biocompatibility, antibacterial properties, electrical conductivity, and cooling functions can be integrated into high‐performance wound dressings, demonstrating significant clinical potential. Based on these developments, we propose the following prospects:

- Multifunctional Integrated Dressings: The development of high‐performance wound dressings that incorporate antimicrobial, conductive and temperature‐regulating functions will be a key research focus. By integrating nanomaterials such as photoluminescent metal nanoclusters and ZnO into polymer matrices, these dressings can offer broad‐spectrum antimicrobial activity, promote tissue regeneration and enable wound cooling under extreme conditions.
- Biodegradable and Sustainable Materials: Bio‐based materials like PLA and CS are expected to see increased use in wound dressings due to their excellent biocompatibility and controlled degradation properties. These materials can eliminate the need for removal and reduce the risks associated with secondary surgeries.
- Clinical Application Research: Strengthen clinical validation of cooling wound dressings in specialised settings such as postsurgical care, chronic wounds, and burns to evaluate their effectiveness in promoting healing and reducing infection.

In summary, the research and development of cooling wound dressings promise to provide more efficient and safer solutions for clinical wound management in the future. Through multidisciplinary collaboration and technological innovation, this field is anticipated to advance further, ultimately benefiting patients.

## AUTHOR CONTRIBUTIONS

Xianhu Liu, Yangzhe Hou, and Jiangang Sun designed this study. Peng Chen and Pingping Zhang contributed equally to this work. All the authors read and approved the manuscrip

## ETHICS STATEMENT

The authors have no competing interest to declare.
